# The Effect of Transcutaneous Electrical Acupoint Stimulation on High-Risk Patients with PONV Undergoing Laparoscopic Gynecologic Surgery: A Randomized Controlled Trial

**DOI:** 10.3390/jcm12031192

**Published:** 2023-02-02

**Authors:** Jiazhu Qin, Xiaoxiao Ye, Changzhou Ye, Xuliang Huang, Huanhuan Sun, Xinyu Zhao, Yao Tong, Manala Mazomba, Yunchang Mo

**Affiliations:** 1 Anesthesia Department, The First Affiliated Hospital of Wenzhou Medical University, Wenzhou 325000, China; 2 Anesthesia Department, Taizhou Hospital of Zhejiang Province, Taizhou 318000, China; 3 Institute of International Education, Wenzhou Medical University, Ouhai District, Wenzhou 325000, China

**Keywords:** transcutaneous electrical acupoint stimulation (TEAS), high-risk patients with PONV, nausea and vomiting, randomized controlled trial

## Abstract

Background: Postoperative nausea and vomiting (PONV) is one of the most common complications after general anesthesia. The traditional comprehensive management of PONV usually uses one or two drugs, but this regimen fails to meet the requirements of the latest version of PONV guidelines. The purpose of this study was to evaluate the effect of transcutaneous electrical acupoint stimulation (TEAS) on high-risk PONV patients who are undergoing laparoscopic gynecological surgery. Methods: In total, 162 high-risk PONV patients were randomly divided into an experimental group (*n* = 81) and a control group (*n* = 81). Both groups were injected with 4 mg of dexamethasone and 0.25 mg of palonosetron. In the experimental group, Nei-guan (PC6) and He-gu (LI4) were stimulated by a transcutaneous acupoint electrical stimulation instrument (HANS200E) 30 min before the surgery. The control group also received electrodes but no stimulation. Variance analysis and rank sum test were used to compare the differences between the two groups. Results: The results of the incidence of postoperative nausea, vomiting, NRS score, degree of abdominal distension, and time to first flatus in the experimental group were lower than those in the control group. Nursing satisfaction of the experimental group was higher than that of the control group. Conclusions: The study demonstrates that TEAS combined with dexamethasone and palonosetron can effectively prevent PONV, reduce postoperative abdominal distension and postoperative pain, and shorten the first postoperative flatus time in high-risk patients with PONV. At the same time, it can improve nursing satisfaction.

## 1. Introduction

PONV (postoperative nausea and vomiting) is one of the most common complications after general anesthesia. When inhaled anesthetics are used without preventive measures, the incidence of PONV after general anesthesia is 30%. In some high-risk patients, the incidence is as high as 70–80% [1]. Different types of surgery can increase PONV, such as gallbladder surgery, laparoscopic surgery, and gynecological surgery [2]. During PONV, the intra-abdominal pressure and central venous pressure of patients increase, and the potential risk of aspiration increases. With the increase in heart rate and blood pressure, the above increases the risk of myocardial ischemia and arrhythmia and increases the postoperative discomfort of patients.

In the General Guidelines for Postoperative Nausea and Vomiting Management in 2020 (Fourth Edition), it is pointed out that adult patients with three or more PONV risk factors are high-risk PONV patients, and at least three to four different types of antiemetics should be used in combination to reduce the proportion of PONV [3]. However, since gynecologic laparoscopic surgery, patients often have high-risk factors of PONV (Apfel score), such as female gender, non-smoking, gynecologic surgery, use of inhalation anesthesia, and use of opioids, the inevitable pneumoperitoneum of laparoscopy and the impact of gynecologic surgery on the digestive tract, the incidence of postoperative nausea and vomiting in gynecologic laparoscopic surgery patients is high [4]. Combinations of drugs were generally more effective than single drugs in preventing vomiting. However, most studies investigated only single drugs, which does not meet the requirements of the latest version of PONV guidelines that at least three or four different types of antiemetics should be used in combination [5], nor does it meet the requirement of improving patient comfort and achieving rapid recovery.

Transcutaneous electrical acupoint stimulation (TEAS) is a technology to prevent and treat diseases by applying a small amount of current close to the bioelectricity of the human body on the surface of the acupoints. It is a therapy combining transcutaneous nerve electrical stimulation with acupuncture and moxibustion. It has the advantages of being simple to use and non-invasive. In recent years, TEAS has been more and more widely used in the clinic, such as in oncology, gynecology and obstetrics, gastrointestinal surgery, cardiac surgery, and other surgical fields [6,7,8]. At present, it has been proven that the application of acupoint stimulation in the perioperative period has good effects on reducing anxiety, sedation and analgesia, organ protection, reduces stress response, reduces postoperative nausea and vomiting. Other research has shown TEAS is associated with a lower cumulative duration of postoperative cognitive dysfunction (POCD) in elderly patients [9]. In this study, PC6 and LI4 were selected. A study showed that PC6 was the pericardial meridian point of hand Jueyin, which was related to the viscera and mainly treated hiccups, vomiting, and stomach pain [10]. LI4 strengthens the spleen and stomach, enriches the blood, and nourishes the meridians of the limbs. Hegu is especially good at treating various limb meridians [11]. At the same time, the combined effect of TEAS and other antiemetic drugs can achieve better efficacy in clinical work. The use of TEAS without increasing the use of drugs can play a similar role in preventing PONV as the antiemetic drugs [4,12]. However, the role of TEAS in high-risk patients with PONV needs to be further explored. Considering the limited influence of drugs on high-risk patients with PONV, and in order to better prevent the occurrence of PONV and improve the postoperative comfort of patients, this study explores the influence of TEAS combined with traditional two-way therapy on high-risk patients with PONV.

### Study Aim

The primary objective of this study was to evaluate the effect of TEAS in preventing postoperative nausea and vomiting in high-risk PONV patients who are undergoing laparoscopic gynecological surgery.

The secondary objective of the study was to evaluate the effect of TEAS on reducing postoperative pain severity, antiemetic drug requirement, and abdominal distension. We also wanted to collect nursing satisfaction after TEAS and the factors related to nursing satisfaction.

## 2. Materials and Methods

### 2.1. Study Design

The study was conducted as a randomized controlled clinical study. A noninterventional control group was used to reveal the difference between TEAS and routine clinical practice. 

### 2.2. Study Setting

The study was conducted at the gynecology department of the First Affiliated Hospital of Wenzhou Medical University from August 2021 to August 2022. This department had 122 beds and a post-anesthesia care unit with 43 beds.

### 2.3. Participants

A total of 162 patients were included in this study, all of whom were diagnosed with uterine leiomyoma, including 92 patients who underwent laparoscopic myoma stripping and 70 patients who underwent a total laparoscopic hysterectomy. Three patients were lost to follow-up during the study. According to the computer random number generator, the patients were divided into an experimental group and a control group, both of which were 81 persons.

#### 2.3.1. Inclusion Criteria

(1) PONV high-risk patients (patients with three or more PONV risk factors: female, non-smoker, gynecological surgery, inhalation anesthesia use, history of motion sickness and opioid use, etc.); (2) patients with a definite diagnosis and requiring laparoscopic surgery; (3) female, aged 18–65 years; (4) American Society of Anesthesiology (ASA) grade I or II; (5) no major organ dysfunction; (6) ECG was normal, and hemodynamics was stable.

#### 2.3.2. Exclusion Criteria

(1) Patients with pacemaker; (2) pregnant patients; (3) language expression or communication difficulties; (4) previous history of central nervous system disease or mental disease or language communication disorder; (5) glucocorticoids, opioids, or antiemetics were used 24 h before operation; (6) those who have received acupuncture treatment in the past; (7) acupuncture site is damaged or infected; (8) status ASA III or higher; (9) local skin infection; (10) patients with upper or lower limb nerve injury; (11) patients who had participated in other clinical trials in the past four weeks; (12) patients with preoperative pain, central analgesics, opioid addiction, and dependence; (13) the researchers considered that they were not suitable to participate in the trial.

#### 2.3.3. Randomization

In the hospital where the study was conducted, laparoscopic gynecological surgery could be performed on more than one patient on the same day. Therefore, randomization should be performed on the day of the surgery to prevent the two groups of patients from influencing each other in the PACU and wards. We used the envelope method for randomization, and each patient was randomly assigned to a group based on surgery day.

### 2.4. Intervention

#### 2.4.1. Anesthesia Protocol

Both groups were treated with tracheal intubation general anesthesia, propofol 2 mg/kg + cisatracurium 0.15 mg/kg + sufentanil 0.4 μg/kg induction, and all patients received propofol, remifentanil, and sevoflurane as intraoperative maintenance drugs. Propofol was maintained at 2.5–5 mg/kg; remifentanil was maintained at 5–10 μg/kg during the operation, according to the patient’s blood pressure and BIS value (aimed for 40–60); an experimental anesthesiologist controlled the dose and recorded the total dose. Because inhaled anesthetics are a risk factor for PONV, we set the MAC of two groups of sevoflurane to 0.3, and the concentration was less than 1%. If the operation lasted more than one hour, we regularly added atracurium 0.03 mg/kg and sufentanil 5 μg per hour. Neostigmine was administered intravenously to reverse the residual effect of the muscle relaxant when the train-of-four (TOF) ratio was less than 0.9 after the operation at 0.05 mg/kg. Before the end of the surgery, all patients were given flurbiprofen axetil 50 mg intravenously for analgesia.

#### 2.4.2. Experimental Methods

In the experimental group, PC6 and LI4 acupoints were stimulated by a transcutaneous acupoint electrical stimulation instrument (HANS200E) 30 min before surgery, with a frequency of 2/100 Hz and a current of 1–12 mA (the stimulation intensity was adjusted according to personal needs, starting from 1 mA and gradually increasing to obtain the maximum tolerance to local muscle slight twitching.). The control group also received electrodes but no stimulation. Both groups were injected with 4 mg of dexamethasone before surgery and 0.25 mg of palonosetron during the surgery.

### 2.5. Outcome

The main outcome was the incidence of nausea and vomiting within 24 h after surgery in the two groups. The secondary outcomes were the number of times the patient vomited, the degree and incidence of nausea, the use of postoperative rescue antiemetic drugs, the level of abdominal distension, the time of first flatus and the NRS score of postoperative pain in the two groups at 2 h, 6 h, and 24 h after operation. At the same time, we record the patient’s age, height, weight, Body Mass Index (BMI), American Society of Anesthesiologists (ASA), Self-Rating Anxiety Scale (SAS), PONV risk factors, surgical methods, intraoperative infusion, anesthesia time, operation time, recovery time of anesthesia and resuscitation room, quality of recovery-40 questionnaire scale (QoR-40), hospitalization time, and hospitalization cost.

### 2.6. Sample Size Calculation

Based on the previous study of patients undergoing laparoscopic gynecological surgery, the incidence rate of PONV was 70%~80% after surgery 24 h. We assumed that it reduced the incidence rate of PONV by 50% TEAS before surgery [13]. Assuming that the difference between the 2 groups would be compared with Chi-square test analysis, using a presumed alpha error of 5% (α = 0.05), a power of 80% (β = 0.2), and considering the potential loss rate of 15%, the sample size was increased to about 160 patients. 

### 2.7. Blinding

The principal investigator of the study received training on TEAS. The study conduct and data collection were carried out by this investigator, and the investigators were not blinded. The patients were informed that they were divided into two groups and could be randomly control or experimental group. The control group received the false electrode patch, and the machine was in working condition, but the electrode wire was disconnected [14]. Therefore, the patient did not know whether they had accepted TEAS. Statistical analysis was performed by a researcher of a member outside of the team, who was blinded to the study.

### 2.8. Data Analyses

Data were evaluated using IBM spss26.0 statistical software. The comparison of patients’ general data, continuous variable data conforming to normal distribution are described in the form of mean and standard deviation (±SD), continuous variable data that were not normally distributed are described in the form of median and interquartile range, and categorical variables are described in specific numbers and percentages. Differences in continuous variables that did not conform to the normal distribution were tested with the Kruskal–Wallis test, and the Bonferroni correction was used to adjust the *p* value for multiple comparisons. The comparison of continuous variable data conforming to normal distribution was presented by ANOVA, and the inter group evaluation of categorical data was presented by Chi-square test or Fisher’s exact test, and the Bonferroni correction was used to adjust the *p* value for multiple comparisons. Linear regression between nursing satisfaction and patient-related data. The *p* value less than 0.05 was considered statistically significant.

## 3. Results

### 3.1. General Information of Patients

There were no significant differences in age, height, weight, BMI, ASA, SAS, PONV risk factors, surgical methods, and other characteristics between the two groups (*p* > 0.05). At the same time, there was no statistical difference in the patients’ intraoperative infusion, anesthesia time, operation time, recovery time of anesthesia and resuscitation room, QoR-40, hospitalization time, and hospitalization cost (*p* > 0.05) (Table 1).

### 3.2. Postoperative Nausea and Vomiting

All patients were followed up for nausea and vomiting within 24 h after operation (Table 2).

Among them, 27 patients (33.33%) developed nausea within 24 h after operation in the experimental group and 43 patients (53.09%) in the control group. The difference between the two groups was statistically significant (*p* = 0.011).

There were 8 patients who vomited (9.90%) in the experimental group and 26 patients (32.10%) in the control group, and the difference between the two groups was statistically significant (*p* < 0.001).

In order to further study the difference between the two groups, the researchers performed subgroup analysis and divided the postoperative 24 h into three periods: 0–2 h, 2–6 h, and 6–24 h. The data were collected by ward nurses and checked by an anesthesiologist at 2, 6, and 24 h after surgery. The nurses asked the patients or guardians and recorded [13]. According to the statistics of the incidence of nausea and vomiting in patients in the three periods, it was found that the patients in the experimental group had less nausea at 0–2 h after the operation, and the difference was statistically significant (3.70% vs. 9.87%, *p* = 0.029). No vomiting occurred in the two groups (experience and control) during this period. The period of high incidence of nausea and vomiting was 2–6 h after surgery. During the 2–6 h period, the incidence of nausea in the experimental group was significantly lower than that in the control group (32.10% vs. 53.09%, *p* = 0.007), and the incidence of vomiting in the experimental group was also lower than that in the control group (8.54% vs. 20%, *p* = 0.014). However, in the 6–24 h was no significant difference in the incidence of nausea between the experimental group and the control group (8.54% vs. 17.5%, *p* = 0.378). There was no significant difference in the incidence of vomiting between the experimental group and the control group (5.00% vs. 6.10%, *p* = 0.732).

### 3.3. Postoperative Pain and Gastrointestinal Condition

This study followed up on the patients’ pain and gastrointestinal recovery at various times after surgery (Table 3).

The pain was evaluated by using Numeric Pain Intensity Scale (NRS), which ranged from 0 (no pain) to 10 (worst possible pain).

Statistics showed that the pain score of the experimental group was no significant difference between the two groups (experimental and control group) 0–2 h (*p* = 0.082) and 2–6 h (*p* = 0.492) after operation. The pain score of the experimental group was also significantly lower than that of the control group at 6–24 h after the operation (*p* = 0.000).

The postoperative gastrointestinal recovery was assessed with the abdominal distension rating scale: Grade 0: the patient had no abdominal distension, and the abdominal circumference increased by <10%; Grade 1: the patient has abdominal distension, and the abdominal circumference increased by <10%; Grade 2: the patient has obvious abdominal distension, and the abdominal circumference increases by 10–20%; Grade 3: pain caused by abdominal distension, abdominal circumference increased by >20%. This study showed that the abdominal distension in the experimental group was lighter than that in the control group at 0–2 h and 2–6 h after surgery (*p* < 0.005); There was no significant difference between the experimental group and the control group in the patients 6–24 h after operation.

### 3.4. Nursing Satisfaction

**Figure 1:**
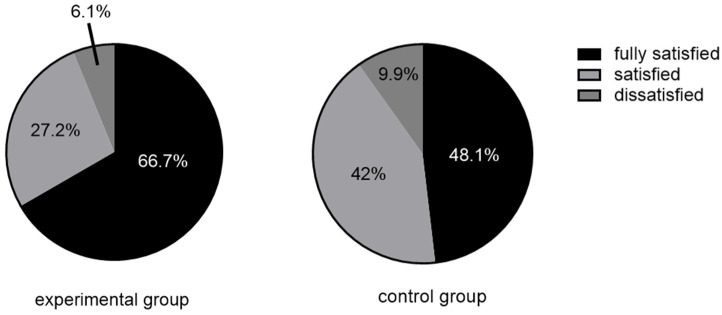
Satisfaction of nursing satisfaction.

This study followed up on the nursing satisfaction of the nurses in charge. Each patient has a nursing satisfaction score table with a score of 0–10 points. The patient fills in their nursing satisfaction when discharged. The study showed that the nursing satisfaction of the experimental group was higher than that of the control group (*p* = 0.028) (Table 4). At the same time, we conducted an analysis of the factors related to nursing satisfaction and found that nursing satisfaction was significantly related to the vomiting and degree of pain of patients (Table 5). According to previous relevant literature, we defined 9–10 as fully satisfied, 6–8 as satisfied, and 1–5 as dissatisfied [15]. The satisfaction result is shown in Figure 1.

## 4. Discussion

### 4.1. Research Meaning

Previous literature shows that PONV is one of the most common and unpleasant complications after general anesthesia. When inhaled anesthetics are used without preventive measures, the incidence of PONV after general anesthesia is 30%. In patients at high risk of PONV, this incidence is as high as 70–80% [1]. In this study, the gynecological patients selected for laparoscopic surgery were all high-risk patients with PONV, and there were four factors affecting PONV, including 1. female, 2. gynecological surgery, 3. inhalation anesthesia use, and 4. opioid use. In this study, the incidence of PONV was 53.09% among the patients who used the combination of palonosetron and dexamethasone to stop vomiting in the control group. It is clear that the current drug treatment to control and prevent postoperative nausea and vomiting can still be improved. Therefore, how to better reduce the incidence of postoperative nausea and vomiting is an important clinical problem that should be solved.

### 4.2. Palonosetron, Dexamethasone, and Transcutaneous Electrical Stimulation of Acupoints

According to the general guidelines for postoperative nausea and vomiting management (Fourth Edition) in 2020, it is recommended to add TEAS therapy combined with palonosetron and dexamethasone for high-risk patients with PONV. Previous literature showed that combined with dexamethasone, electrical acupoint stimulation or tropisetron is more effective in PONV prophylaxis than dexamethasone alone in gynecological patients undergoing laparoscopic surgery [16]. Our study found that 27 patients (33.33%) in the experimental group developed nausea within 24 h after the operation, which was significantly less than 43 patients (53.09%) in the control group. Among the patients who vomited within 24 h after the operation, 8 cases (9.90%) in the experimental group were significantly less than 26 cases (32.10%) in the control group. It is suggested that the combination of TEAS and traditional dual drugs can effectively reduce the incidence of postoperative nausea and vomiting in high-risk patients with PONV.

Previous studies have shown that the incidence of PONV in patients after laparoscopic surgery is related to the release of 5-HT3 by gastrointestinal chromaffin cells and the activation of 5-HT3 receptors under gastrointestinal mucosa caused by surgical stimulation [17]. Palonosetron has a high affinity and high selectivity and is a long-acting 5-HT receptor antagonist. It was first used to prevent nausea and vomiting caused by chemotherapy, and later widely used to prevent PONV, and was written into relevant guidelines [18]. Campos et al. found that Palonosetron exhibited efficacy in reducing the overall incidence of PONV after TAH under spinal anesthesia with intrathecal morphine [19]. Dexamethasone is a kind of corticosteroid that can inhibit the synthesis of prostaglandins, promote the release of endorphins, and prevent nausea and vomiting [20]. The effect of preventing postoperative nausea and vomiting is significant, and the action time can be as long as 6–12 h [21].

Transcutaneous electrical stimulation of acupoints is a non-invasive and convenient operation that can even be bought and operated by oneself. At present, TEAS has been widely used in many disciplines, and TEAS can reduce anxiety, provide sedation and analgesia, protect organs [22], prevent preinduction hypertension in patients [23], diminish the upregulation of proinflammatory factors [24], and reduce the incidence of PONV, PON, and POV [25]. It has been found that acupuncture at PC6 has a significant effect on preventing nausea and vomiting. The mechanism may be by affecting the neuroendocrine system, such as promoting the release of endogenous morphine-like substances and activating adrenergic and noradrenergic nerve fibers to change the transmission of 5-hydroxytryptamine [26]. TEAS can be comparable to antiemetics in preventing nausea and vomiting [1]. However, a study did not find the stimulation of the PC6 acupoint with an acupressure wristband to be clinically effective in reducing postoperative nausea and vomiting or antiemetic drug requirement [27]. Another study indicated that PC6 acupressure has the short-term effect of relieving nausea but not vomiting and retching [28]. The application of TEAS in gynecological laparoscopic surgery was found to significantly promote the quality of early recovery, improve MMSE scores, and reduce the incidence of pain, nausea, and vomiting in patients [29]. The effectiveness of acupoint stimulation needs further study.

### 4.3. PONV

In order to further study the difference in PONV between the two groups, the researchers performed subgroup analysis and divided the postoperative 24 h into three periods: 0–2 h, 2–6 h, and 6–12 h. According to the statistics of the number of patients with nausea and vomiting in both periods, it was found that in postoperative 0–2 h, the number of patients with nausea in the experimental group (3.70%) was significantly lower than that in the control group (9.87%), Vomiting did not occur in both groups. This was attributed to the fact that the patient had just immediately finished surgery and was in the early stage of recovery. The anesthetic drugs had not been completely metabolized, and the gastrointestinal function and senses were not recovered. Therefore, PONV occurs less.

From two to six hours after the operation was the period of high incidence of nausea and vomiting. Regarding nausea, there were 26 patients (32.10%) in the experimental group and 43 patients (53.09%) in the control group. There was a significant difference between the two groups. The number of patients who vomited in the experimental group (7 cases) (8.54%) was significantly lower than that in the control group (16 cases) (20%), and 2–6 h after the operation, the patient’s narcotic drug metabolism was complete and began to recover gradually. The gastrointestinal tract began to peristalsis, and the stress response gradually appeared. This study showed that the incidence of PONV was the highest at this time. However, Xiong et al. [13] showed that the incidence of PONV was the highest 6–12 h after surgery (71%), followed by 2–6 h (51.6%). This may be because our object of study is patient underlying laparoscopic gynecological surgery, while Xiong studied female patients undergoing laparoscopic sleeve gastrectomy. There were also differences in surgery time, patient BMI, and postoperative opioids. These different factors may account for the difference in PONV high-risk period. In our study, the incidence of the experimental group was significantly lower than that of the control group, indicating that the combination of TEAS with panosetron and desamethasone has a better effect on the prevention of PONV.

Nausea occurred in 7 cases (8.54%) in the experimental group and 14 cases (17.5%) in the control group 6–24 h after the operation. There was no significant difference between the two groups; there were 4 cases (5.00%) of vomiting in the experimental group and 5 cases (6.10%) of vomiting in the control group, and there was no statistical difference. The number of patients with PONV decreased gradually after surgery, and the number of patients in the experimental group was less than that in the control group. The difference was not significant, indicating that most patients recovered well at this time. This shows that TEAS combined with panosetron and dexamethasone has no significant long-term effect on PONV. A meta-analysis showed that PC6 acupuncture significantly reduced the number of cases of early vomiting (postoperative 0–6 h) and nausea (postoperative 0–24 h) but not early nausea (postoperative 0–6 h) and vomiting (postoperative 0–24 h [30]. In our study, the number of early nausea (postoperative 0–6 h) in the experimental group was significantly lower than that in the control group (*p* = 0.027), so this was inconsistent with the results in this paper. However, the results of the two articles were consistent in terms of postoperative vomiting. It may also be caused by insufficient sample size, and further research is needed.

### 4.4. Postoperative Pain

Perioperative pain is one of the biggest problems affecting the rehabilitation of patients. Pain is not only not conducive to circulation stability but also increases the risk of complications. High-dose opioids can effectively control pain but may cause respiratory depression. The analgesic effect of TEAS is widely used in the perioperative period. It may play a role through peripheral, spinal, and supraspinal mechanisms [31]. Previous studies found that acupuncture at LI4 and PC6 can stimulate the secretion of central opioid peptide, further hinder the upward transmission of pain information in the spinal thalamic tract, and enhance the postoperative analgesia effect [25]. Liu et al. [32] found that electrical stimulation before anesthesia induction can significantly reduce the pain level of patients 24 h after the operation, improve the postoperative comfort of patients and improve the prognosis. Maimer et al. [33] found that acupoint therapy has a significant analgesic effect after sternotomy; it is not only effective for acute pain but also has been shown that TEAS can reduce the chronic pain of mastectomy lasting up to 6 months [34]. Our study showed that the pain in the experimental group was lower than that in the control group after 6–24 h, with statistical significance. There was no difference between the two groups for 0–2 and 2–6 h. It can be seen that TEAS can relieve the postoperative pain of high-risk patients with PONV for a long duration, and there is still a significant effect even 24 h after the operation. However, it should be noted that 6–24 h NRS (0.61 ± 0.85 vs. 0.96 ± 1.87, *p* < 0.001), this value is statistically significant, but it may not have clinical significance due to the close score and the subjective data of patients. 

In this study, there was no significant difference between the two groups in postoperative pain 0–2 h and 2–6 h after the operation. Considering that the anesthetic was not completely metabolized or the patient had just moved from the operating room to the ward, there was a postural change that may affect the occurrence of postoperative pain. Previous studies found that acupuncture at LI4 and PC6 can stimulate the secretion of central opioid peptide, further hinder the upward transmission of pain information in the spinal thalamic tract, and enhance the postoperative analgesia effect [25]. The conclusion is consistent with this study.

### 4.5. Gastrointestinal Recovery

In terms of gastrointestinal recovery, the study found that the abdominal distension of the experimental group was better than that of the control group at 0–2 h and 2–6 h after the operation; there was no significant difference between the two groups at 6–24 h after operation. The incidence of abdominal distension was consistent with the incidence of PONV in the two groups. The incidence in the experimental group was lower than that in the control group at 0–6 h, and there was no significant difference between the two groups at 6–24 h. Traditional Chinese medicine believes that stimulation of PC6 can reduce gastrointestinal pressure, stop nausea, and promote gastrointestinal peristalsis [35]. Therefore, abdominal distension, nausea, and vomiting belong to gastrointestinal reactions and are closely related. In the comparison of postoperative time to the first flatus at 0–2 h, 2–6 h, and 6–24 h, it was found that the time to the first flatus of the experimental group was significantly earlier than that of the control group. It indicates that electrical stimulation can shorten the postoperative time to the first flatus and promote gastrointestinal peristalsis. Therefore, TEAS can not only relieve the postoperative pain of patients but also promote the recovery of gastrointestinal function.

### 4.6. Nursing Satisfaction

This paper found that TEAS combined with drug therapy can improve nursing satisfaction. Through analysis of related factors, it was found that nursing satisfaction was mainly related to patients’ vomiting and pain. This is consistent with previous research results [36]. It is mainly severe pain and vomiting that will bring great discomfort to the patient. It is necessary to call nurses to provide additional auxiliary measures beyond routine treatment. However, this problem would increase clinical work and may lead to doctor–patient conflicts. The use of TEAS combined with drugs can reduce the discomfort of patients and improve their satisfaction with nursing.

### 4.7. Limitation

The research also has some limitations. (1) The research had a single gender that only included gynecological female patients, which means that all patients at high risk of PONV were not included. (2) The research is a single-center experiment, which requires more convincing data from multiple centers in the future. (3) This research found that the incidence of nausea and vomiting and the time to first flatus in the experimental group were less than those in the control group in 0–6 h, and the pain in 6–24 h was less than that in the control group. However, there was no significant difference between the two groups in the quality of recovery scale after surgery (PQRS). This may be because the patient has no time limit when filling in the form. Patients always fill in the form when they feel good. (4) Due to ethical rejection, this article does not include a placebo group and a control group with acupuncture treatment alone.

## 5. Conclusions

In summary, the results of this study show that preoperative TEAS combined with intraoperative use of dexamethasone and palonosetron can effectively prevent postoperative nausea and vomiting in high-risk patients with PONV, reduce postoperative abdominal distension, shorten the first postoperative time to first flatus and postoperative pain, increase the postoperative comfort of patients, increase nursing satisfaction, and improve the postoperative recovery quality of patients. The formulation and implementation of the PONV management strategy need to consider the cost–benefit ratio of treatment and the availability of drugs. At present, the research on the combination of TEAS and multiple antiemetics is still under constant exploration. It can be determined that the treatment of PONV by acupoint stimulation is effective, safe, and low-cost and has been included in the international PONV management guidelines as the only non-drug intervention.

## Figures and Tables

**Table 1:** Patient baseline characteristics and perioperative variables.

	Experience Group (81)	Control Group (81)	*p*
Age (years) mean ± SD	45 ± 7.45	45 ± 5.92	0.494
Height (m) mean ± SD	1.58 ± 0.47	1.59 ± 0.54	0.321
Weight (kg)mean ± SD	57.27 ± 10.43	58.61 ± 7.06	0.167
BMI ^1^ (kg/m^2^) mean ± SD	23.25 ± 2.69	23.13 ± 2.72	0.243
SAS ^2^ (mean ± SD)	29.30 ± 3.87	28.52 ± 4.45	0.611
PQRS ^3^ (mean ± SD)	176.04 ± 6.23	175.00 ± 6.14	0.736
Hospital stay (days) mean ± SD	5.87 ± 1.75	5.43 ± 1.62	0.165
Postoperative hospital stay (days) mean ± SD	3.41 ± 1.41	3.48 ± 1.38	0.997
Hospitalization expenses (dollars) mean ± SD	2412.83 ± 371.84	2530.05 ± 512.95	0.473
Intraoperative infusion (mL) M (p_25_,p_75_)	800 (600,1000)	900 (600,1100)	0.217
Anesthesia Duration (min) M (p_25_,p_75_)	110 (79.5,124.5)	111.5 (94.75,140)	0.147
Operation duration (min) M (p_25_,p_75_)	85 (65.75,100)	86 (73.5,113.25)	0.066
Recovery time M (p_25_,p_75_)	52 (45,61)	45 (41,52.25)	0.086
ASA ^4^ (I/II)	29/52	31/50	0.121
Operation mode (n) (Myomectomy/Hysterectomy)	41/40	51/30	0.221
Smoker (n)	0/81	0/81	/
Motion sickness (n)	1/81	1/81	/

^1^ BMI: Body Mass Index; ^2^ SAS: Self-Rating Anxiety Scale; ^3^ PQRS: Postoperative Quality Recovery Scale; ^4^ ASA: American Society of Anesthesiologists.

**Table 2:** Comparison of nausea and vomiting between experimental group and control group.

		Experience Group	Control Group	*p*
Nausea	0–24 h (n,%)	27 (33.33%)	43 (53.09%)	0.011 *
	0–2 h (n,%)	3 (3.70%)	8 (9.87%)	0.029 *
	2–6 h (n,%)	26 (32.10%)	43 (53.09%)	0.007 *
	6–24 h (n,%)	7 (8.54%)	14 (17.5%)	0.378
Vomiting	0–24 h (n,%)	8 (9.90%)	26 (32.10%)	0.000 *
	0–2 h (n,%)	0 (/)	0 (/)	/
	2–6 h (n,%)	7 (8.54%)	16 (20%)	0.014 *
	6–24 h (n,%)	4 (5.00%)	5 (6.10%)	0.732

* Significant difference at alpha < 0.05.

**Table 3:** Comparison of postoperative pain, abdominal distension, and time to first flatus.

Postoperative	Experimental Group	Control Group	*p*
0–2 h NRS ^1^ (mean ± SD)	0.99 ± 0.71	1.19 ± 1.11	0.082
2–6 h NRS (mean ± SD)	1.56 ± 1.58	1.7 ± 2.03	0.492
6–24 h NRS (mean ± SD)	0.61 ± 0.85	0.96 ± 1.87	0.000 *
Abdominal distension 0–2 h (Grade 0/1/2/3)	64/17/0/0	37/44/0/0	0.000 *
Abdominal distension 2–6 h (Grade 0/1/2/3)	41/38/2/0	26/45/7/3	0.000 *
Abdominal distension 6–24 h (Grade 0/1/2/3)	48/33/0/0	50/31/0/0	0.793
Time to first flatus (min) mean ± SD	1063.87 ± 290.07	1254.50 ± 386.89	0.048 *

* Significant difference at alpha < 0.05. ^1^ NRS: Numeric Pain Intensity Scale.

**Table 4:** Comparison of nursing satisfaction between the experimental group and the control group.

	Experimental Group	Control Group	*p*
Nursing satisfaction (mean ± SD)	8.71 ± 1.32	8.37 ± 1.37	0.029 *

* Significant difference at alpha < 0.05.

**Table 5:** Analysis of the factors related to nursing satisfaction.

	B	Std. Err.	Beta	t	*p*
Nausea	0.013	0.145	0.005	0.089	0.929
Vomiting	−1.626	0.182	−0.493	−8.941	0.000 *
Marked nausea	−0.426	0.233	−0.101	−1.829	0.069
Degree of pain	−0.333	0.034	−0.519	−9.823	0.000 *

* Significant difference at alpha < 0.05.

## Data Availability

The datasets used and analyzed during the current study are available from the corresponding author upon reasonable request.
